# Melanoma biology and treatment: a review of novel regulated cell death-based approaches

**DOI:** 10.1186/s12935-024-03220-9

**Published:** 2024-02-09

**Authors:** Ming-yun Hsieh, Sheng-Kai Hsu, Tzu-Yu Liu, Chang-Yi Wu, Chien-Chih Chiu

**Affiliations:** 1 Department of Pediatrics, Kaohsiung Veterans General Hospital, Kaohsiung, Taiwan; 2 Department of Biological Sciences, National Sun Yat-Sen University, Kaohsiung, 804 Taiwan; 3 Department of Biotechnology, Kaohsiung Medical University, Kaohsiung, 807 Taiwan; 4 Center for Cancer Research, Kaohsiung Medical University, Kaohsiung, 807 Taiwan; 5 Department of Medical Laboratory Science and Biotechnology, Kaohsiung Medical University, Kaohsiung, 807 Taiwan; 6 Department of Medical Research, Kaohsiung Medical University Hospital, Kaohsiung, 807 Taiwan

**Keywords:** Melanoma, Regulated cell death, Autophagy-dependent cell death, Necroptosis, Ferroptosis, Pyroptosis, Cuproptosis, Immunotherapy, Targeted therapy

## Abstract

The incidence of melanoma, the most lethal form of skin cancer, has increased due to ultraviolet exposure. The treatment of advanced melanoma, particularly metastatic cases, remains challenging with poor outcomes. Targeted therapies involving BRAF/MEK inhibitors and immunotherapy based on anti-PD1/anti-CTLA4 antibodies have achieved long-term survival rates of approximately 50% for patients with advanced melanoma. However, therapy resistance and inadequate treatment response continue to hinder further breakthroughs in treatments that increase survival rates. This review provides an introduction to the molecular-level pathogenesis of melanoma and offers an overview of current treatment options and their limitations. Cells can die by either accidental or regulated cell death (RCD). RCD is an orderly cell death controlled by a variety of macromolecules to maintain the stability of the internal environment. Since the uncontrolled proliferation of tumor cells requires evasion of RCD programs, inducing the RCD of melanoma cells may be a treatment strategy. This review summarizes studies on various types of nonapoptotic RCDs, such as autophagy-dependent cell death, necroptosis, ferroptosis, pyroptosis, and the recently discovered cuproptosis, in the context of melanoma. The relationships between these RCDs and melanoma are examined, and the interplay between these RCDs and immunotherapy or targeted therapy in patients with melanoma is discussed. Given the findings demonstrating melanoma cell death in response to different stimuli associated with these RCDs, the induction of RCD shows promise as an integral component of treatment strategies for melanoma.

## Introduction

Melanoma, a life-threatening malignancy primarily affecting the skin, affects different primary sites, with cutaneous, ocular, and mucosal sites accounting for 93.3%, 5.5%, and 1.3% of reported cases, respectively [1]. The 5-year survival rate for all stages of cutaneous melanoma has been reported to be 94% by the Surveillance, Epidemiology, and End Results (SEER) Program. In contrast, survival rates for metastatic cutaneous melanoma were significantly lower, at 39.4% diagnosed between 2016 and 2018 [2]. In a study involving 428 patients with metastatic melanoma treated with checkpoint inhibitors from 2007 to 2018, the 5-year survival rates for cutaneous, acral, uveal (ocular), and mucosal melanoma were 46%, 34%, 21%, and 22%, respectively [3]. The various prognoses of these subtypes reflect distinct pathogenesis and genetic alterations. Understanding the underlying pathogenesis is crucial for overcoming treatment obstacles in unresponsive metastatic melanomas. In the first part of this article, we provide an overview of the pathogenesis and current treatment options and limitations for melanoma.

In the second part of the article, the relationship between regulated cell death (RCD), namely, autophagy-dependent cell death, pyroptosis, necroptosis, ferroptosis and cuproptosis, and melanoma is discussed. Autophagy-dependent cell death strictly requires autophagy induction. Pyroptosis is a proinflammatory type of RCD characterized by apoptotic body-like protrusions on the plasma membrane established during gasdermin pore formation. Necroptosis is regulated by various cytokines and pattern recognition receptors (PRRs). Ferroptosis specifically depends on high intracellular iron concentrations and is characterized by the accumulation of lipid peroxides. Cuproptosis is a copper-dependent form of cell death regulated by mitochondrial ferredoxin 1 (FDX1)-mediated protein lipoylation. All these RCDs differ from apoptosis and have been studied in the melanoma context, either in vitro or in vivo, showing a promising role in inducing the death of melanoma cells. This review provides a comprehensive overview of these RCDs and their interactions with BRAF/MEK inhibitors or immune blocker therapy. Since the current treatment choice for melanoma, including BRAF/MEK inhibitors or check blocker therapy, has drawbacks, understanding these nonapoptotic RCDs may help to identify new therapeutic targets for melanoma treatment.

## Pathogenesis and treatment limitations of melanoma

### Overview of pathogenesis

Melanoma is a malignancy originating from melanocytes located in the basal layer of the epidermis. Because melanocytes are also in the digestive tract, urogenital tract and mucous glands, noncutaneous melanoma can affect these tissues, and noncutaneous melanoma represents approximately 5% of all cases of melanoma [4]. Melanomas may develop in or near a previously existing precursor lesion, including a common nevus, dysplastic nevus, congenital nevus, and blue nevus, or in healthy-appearing skin. It has two growth phases: radial and vertical phases. When melanoma tumors are thin, superficial and primarily confined to the epidermis, they are in the radial growth phase and indolent. With the development of the vertical growth phase, which can arise de novo or from lesions in the radial growth phase, melanoma cells invade deep into tissues and show metastatic potential.

The incidence of melanoma is increasing worldwide in white populations, especially where fair-skinned people receive excessive sun exposure; for example, 20–30 cases per 100,000 individuals per year are reported in the United States, and 50–60 cases per 100,000 individuals per year are reported in Australia; in contrast, the melanoma incidence is lower than 5 cases per 100,000 individuals per year in Asia and Africa [5, 6]. Excessive sun exposure, indoor tanning booths, the number of typical nevi, the presence of atypical nevi, a personal history of melanoma, and a family history of melanoma all increase the risk of melanoma. Traditionally, invasive cutaneous melanoma is morphologically classified into 4 subtypes: superficial spreading melanoma, nodular melanoma, lentigo malignant melanoma, and acral lentiginous melanoma [7]. With the discovery of genetic alterations in melanocytic tumors, the new classification incorporates the clinical, pathological, and genomic characteristics of melanoma. Therefore, the 2018 World Health Organization (WHO) classification of melanocytic tumors indicates nine evolutionary pathways into three major categories of melanomas as determined by the intensity of chronic ultraviolet radiation exposure/cumulative solar damage, ranging from low cumulative solar damage (CSD)-related (pathway 1), high CSD-related (pathways 2 and 3), and non-CSD-related melanoma (pathways 4–9) [8, 9] (Table 1). In addition, the American Joint Committee on Cancer (AJCC) staging system, which is based on tumor size, regional involvement of lymph nodes, and distant metastasis, is commonly used in clinical practice to record disease severity, guide treatment options, and predict prognosis [10].

**Table 1:** The classification of melanomas (by 2018 WHO Classification) and associated genomic alterations

Evolutionary pathway	UVR exposure/CSD	Melanoma subtype/ anatomical location	Affected genes				
			MAPK pathway	Cell cycle	Telomerase pathway	PI3K/AKT pathway	Chromosomal aberrations
I	Low UVR exposure/CSD	Superficial spreading melanoma (SSM)/ skin	BRAF V600 (45–50%) [8]: E (80%)/K (15%) [9] ; NRAS (30%); NF1(15%), triple wild type (WT) (5–10%); KIT (5–10%) [8]	CDKN2A (~ 13–40%: mutation; ~ 45%: loss); TP53(~ 15–18%) [8]	TERT (mutation or gain): 85% [8]	PTEN (8.5–40%) [8]	Low [8]
II	High UVR exposure/CSD	Lentigo malignant melanoma (LMM)/ skin	NRAS (~ 14%) [8]; BRAF (~ 22%) [11]; KIT(~ 10%) [12]	Also mutations in CCND1, MITF, and p53 [13]			
III		Desmoplastic melanoma/ skin	BRAF (0–5%); RAS (0–6%); NF1 (52–93%); triple WT (7–48%); KIT (rare) [8]	CDKN2A (20–29%: mutation; 18%: loss); TP53 (40–60%) [8]	TERT (mutation or gain: 85%) [8]	PTEN (rare) [8]	Low [8]
IV	Low to no UVR exposure/CSD	Spitz melanoma/ skin	Spitz associated genetic change include *HRAS* or *MAP2K1* mutations, copy number gains of 11p, and fusions involving *ALK, ROS, NTRK1, NTRK2, NTRK3, MET, RET, MAP3K8,* and *BRAF* genes [14]				
V		Acral melanoma/ palms, soles or nail beds	BRAF V600 (10–35%); NRAS (8–22%); NF1 (11–23%); triple WT(45–58%); KIT (3–36%) [8]	CDKN2A (0–3%: mutation; 35%: loss); TP53 (6–54%) [8]	TRET (mutation or gain: 9–45%) [8]	PTEN (26–28%) [8]	High [8]
VI		Mucosal melanoma/ mucosa of respiratory, gastrointestinal or urogenital tract	BRAF: 0–21%, RAS: 5–25%, NF1 (0–18%), triple WT: 65–75%, KIT: 7–25% [8]	CDKN2A (rare mutation; 10–38%: loss); TP53: 7–15% [8]	TERT (mutation or gain: 5–13%) [8]	PTEN (4–25%) [8]	High [8]
VII		Melanoma arising in congenital nevus/ skin	BRAF: < 30% mutation, possible fusion [15, 16] NRAS 80–95% [17]	TP53 (rare) [18]	TERT (hypermethylation or gain: case report) [146]	PTEN (proposed role to stop melanoma transformation) [147]	High [17]
VIII		Melanoma arising in blue nevus/ skin	*Common GNAQ* and *GNA11* ***mutations***, occasional *PLCB4* or *CYSLTR2* mutations, less *BRAF* or other mutations associated with melanoma, not high TMB [19, 20]				
IX		Uveal melanoma/ iris, ciliary body or choroid	Rare BRAF, RAS, and NF1 mutation, triple WT (~ 100%), KIT (11%) [8]	CDKN2A (rare mutation, 12%: loss); TP53 (9%) [8]	TERT (mutation or gain: 5–13%) [8]	PTEN (∼6–11%) [8]	High [8]

The WHO classification of melanoma not only reflects the pathway concept of melanoma pathogenesis but also reveals distinct molecular signatures associated with different anatomical locations and levels of patient sun exposure. Melanoma lesions associated with low ultraviolet (UV) exposure/CSD (pathway 1) are located mainly on the trunk and extremities, and approximately 45 ~ 50% of these tumors carry a BRAF mutation (Table 1) [6, 8]. Melanoma lesions associated with high UV exposure/CSD (pathways 2 and 3) are located mainly in the head and neck region and show a moderate frequency of NRAS (NRAS is a proto-oncogene and a GTPase) mutations, which are found in approximately 10–20% of cutaneous melanomas [21]. Non-sun-related melanoma lesions (pathways 4 to 9) are located mainly on acral and mucosal sites and do not carry BRAF, NRAS, or neurofibromin 1 (NF1) mutations (triple wild-type) and exhibit a low frequency of c-KIT (KIT proto-oncogene, receptor tyrosine kinase) mutations (15%) [9, 22]. All BRAF, NRAS and NF1 mutations can activate the mitogen-activated protein kinase (MAPK) pathway and are generally introduced in the early stages of tumor evolution as driver mutations [23]. In addition to these driver mutations, a combination of genetic alterations leads to cancer development. In cutaneous melanoma, subsequent mutations in the telomerase reverse transcriptase (TERT) promoter and in regulators of the cell cycle, such as cyclin-dependent kinase inhibitor 2A (CDKN2A), are introduced before the mutation of TP53, which is associated with advanced stages of primary tumor progression [23].

### Current treatment options and limitations

Approximately 90% of melanomas are diagnosed as primary tumors without any evidence of metastasis, with a tumor-specific 10-year survival of 75–95% [6]. For primary melanoma without metastasis, excision with a safety margin remains the standard of care [24]. Sentinel lymph node dissection should be performed as a staging procedure in patients with tumor thickness ≥ 1.0 mm or ≥ 0.8 mm with additional histological risk factors [24]. Positive lymph node involvement of melanoma is classified as at least stage III disease. For melanoma with distant metastasis (stage IV) irrespective of local tumor resectability and stage III melanoma, systemic therapy is proposed [24]. However, before 2010, no randomized controlled trial had demonstrated a survival advantage in people with advanced melanoma, and the median overall survival for patients with stage IV melanoma was less than one year [25]. At that time, chemotherapy was the only systemic treatment for metastatic melanoma, with a low response rate of 12.1–17.6% for dacarbazine [26–29], the only U.S. Food and Drug Administration (FDA)-approved chemotherapy for melanoma. In contrast, chemotherapy is now considered the last-line treatment and is used in patients with resistance to immunotherapy and targeted therapy. To date, the first-line systemic therapy has been immunotherapy, regardless of BRAF mutational status, or alternatively, a combination of BRAF and MEK inhibitors for patients carrying a BRAF mutation [24].

Immunotherapy exploits one’s own personal immune system to kill cancer cells. Since the FDA approval of ipilimumab (anti-cytotoxic T-lymphocyte-associated protein 4 (CTLA4) antibody) to treat metastatic melanoma in 2011 [30], immune checkpoint blocker therapy has represented the primary immunotherapy for melanoma, as it targets CTLA4 and PD-1 on T cells or programmed cell death ligand 1 (PD-L1) on tumor cells, enabling them to escape antitumor responses [24]. Established to evaluate the combined use of anti-PD1 and anti-CTLA4 antibodies for advanced cutaneous melanoma, the phase IIIb/IV CheckMate 511 study reported a 53% response rate and a 61% 3-year overall survival rate [31]. Another trial, CheckMate 067, evaluating the effects of nivolumab (an anti-PD1 antibody) plus ipilimumab and nivolumab alone *versus* ipilimumab alone for patients with previously untreated unresectable stage III or stage IV melanoma showed 6.5-year overall survival rates of 57%, 43%, and 25% in patients with BRAF-mutant melanoma and 46%, 42%, and 22% in those with BRAF-wild-type melanoma, respectively [32]. From the above experience, it is suggested that the combined use of anti-PD1 and anti-CTLA4 antibodies yields a better response than the solitary use of either antibody, and approximately half of patients at an advanced stage of melanoma can benefit long-term from immunotherapy.

In patients carrying the BRAF V600E mutation, the combination of BRAF and MEK inhibitors such as vemurafenib (a BRAF inhibitor) and cobimetinib (a MEK inhibitor) resulted in a better response than BRAF inhibitors alone and led to a treatment response in a small subset of patients with disease progression receiving BRAF inhibitors alone. Compared to the 53% response rate to vemurafenib alone reported by Sosman et al. [33], an 87% response rate in patients who had never received a BRAF inhibitor and a 15% response rate in patients whose disease had recently progressed while taking vemurafenib alone were recorded in a trial to evaluate the combined use of vemurafenib and cobimetinib [34]. Despite the improved response rate of combined use of BRAF and MEK inhibitors compared with single use, the combination of BRAF and MEK inhibitors for advanced melanoma in patients carrying the BFAF V600E mutation yielded a 50–70% response rate but an approximately 30% 5-year survival rate [24, 34, 35].

Apart from unsatisfactory long-term survival by either immunotherapy or targeted therapy, the treatment options for some types of melanomas, including acral melanoma, mucosal melanoma and uveal melanoma, remain very limited. Due to ill-defined tumor margins, local recurrence often occurs after surgery in patients with acral and mucosal melanoma [36]. Despite effective local therapy, nearly 50% of patients with uveal melanoma develop metastatic disease [37]. In addition, in acral melanoma, the BRAF V600 mutation rate is 10–35%, which limits the use of BRAF/MEK inhibitors (Table 1). C-Kit inhibitors such as imatinib used for metastatic melanomas, mostly from acral and mucosal melanoma with c-KIT alterations, showed an overall response rate of 23% [38]. In addition to uncommon BRAF and c-KIT mutations (Table 1), the unsatisfactory response to currently available targeted therapy with BRAF and c-KIT inhibitors, the paucity of large studies and the rarity of these subtypes also restrict their standard use in patients with acral, mucosal or uveal melanoma [36, 39]. Furthermore, the tumor mutation burden (TMB) of these subtypes of melanoma was usually lower than that of cutaneous melanoma, corresponding to no CSD in the genetic pathology, such that the response to immune checkpoint blocker therapy was unsatisfactory, with a 15.6–43% response rate to the combined use of anti-PD1 and anti-CTLA4 antibodies [40, 41]. In addition, considering that melanoma at AJCC low-risk stages I and IIA accounts for most of the deaths because of the high number of patients with this diagnosis and some so-called high-risk patients with stage III or IIB-C disease who are exposed to systemic treatment but do not need it, finding a better biomarker linked to prognosis is necessary [24].

Therefore, to increase the response rate of immune checkpoint blocker therapy, nivolumab (anti-PD1 antibody) plus an anti-lymphocyte-activation gene 3 (LAG3) antibody (relatlimab) received approval in the United States for the treatment of unresectable or metastatic melanoma in adult patients in March 2022 [42]. Tebentafusp, a bispecific antibody that targets the glycoprotein 100 (gp100) protein on tumor cells and CD3 on T cells, received approval for the treatment of unresectable or metastatic uveal melanoma in January 2022 [43]. The first viral oncolytic vaccine, talimogene-laherparepvec (T-VEC), was even approved by the FDA as early as 2015 for patients with unresectable metastatic melanoma with improved local control but a mild systemic effect [44]. The ideal implantation of combined BRAF/MEK inhibitors and immune checkpoint blocker therapy, the involvement of oncolytic viral vaccines with immunotherapy, new drug development and nanotechnology-based administration systems for melanoma treatment are ongoing [45]. The development of novel treatments for targeting biomarkers responsible for melanoma progression, as alternatives or as complementary treatments to immunotherapy, is an outcome of recent investigations [46]. One strategy for using potential novel agents is the induction of RCD. Different types of RCD can affect cancer progression and response to therapy, and evasion from RCD is one of the important characteristics of cancer cells[47]. Furthermore, RCD is essential for controlling the immunosuppressive tumor microenvironment (TME) [48]. In addition, developing a RCD-related gene expression score for use as a reliable biomarker to predict prognosis and guide treatment choices is a possible strategy to offer personalized therapy [49].

## Regulated cell death in the melanoma context

RCD plays a critical role in cell homeostasis, tissue remodeling, and tumorigenesis. Apoptosis is the most well-characterized type of RCD and is the major RCD modality of cancer cells. Clinically, many traditional chemotherapeutic agents have been designed to induce apoptosis in cancer cells. Therefore, evasion from apoptosis not only enables defective cells with dangerous mutations to undergo tumorigenesis but also promotes therapy resistance. With the discovery of more types of RCD, it is now evident that cancer cells can succumb to other RCD pathways in addition to apoptosis. Research on nonapoptotic pathways of RCD as alternative therapeutic targets is an attractive option to overcome the failure of apoptosis induction in cancer cells. The importance of nonapoptotic cell death signaling pathways, such as autophagy-dependent cell death, necroptosis, ferroptosis, pyroptosis, and cuproptosis, in melanoma formation and progression is discussed in the following section. In addition, the connections among these RCDs, melanoma and immunotherapy are discussed.

### Autophagy-dependent cell death

**Fig. 1:**
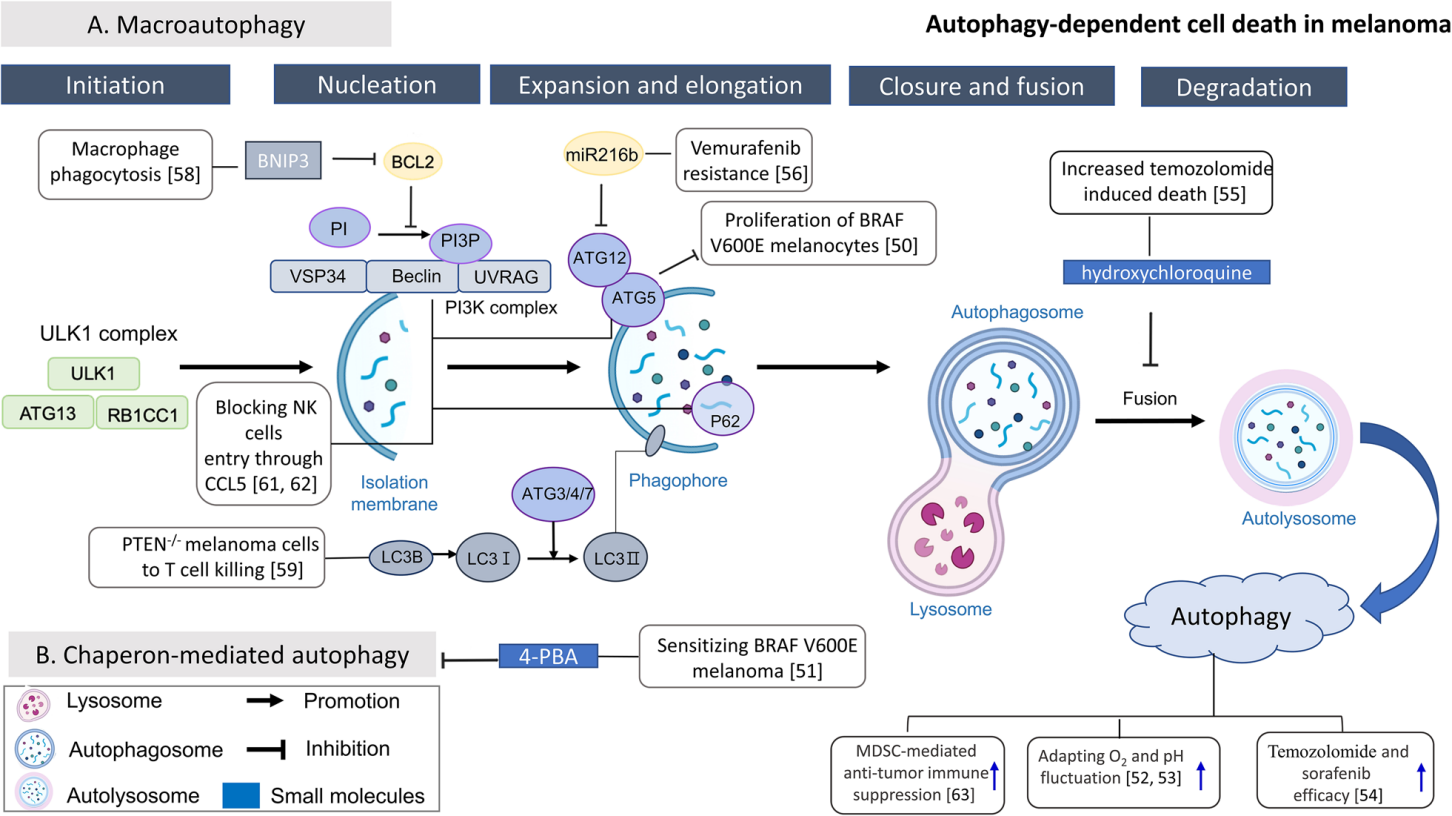
Schematic diagram of autophagy-dependent cell death and related research in melanoma. Autophagy is a process initiated by ULK1 complex formation, followed by membrane isolation and phagospore formation. After fusion with lysosome, autophagosome ends up and engulfed substrates are digested in autophagosome. Related researches are expressed in white squares with reference number in parenthesis. The exogenous small molecules are marked in blue squares especially. For example, vemurafenib (a BRAF inhibitor) resistance is associated with elevated expression of Beclin-1, ATG5 and UVRAG, and its efficacy can be increased by ectopic expression of miR-216b to inhibit ATG5. The manipulation of autophagy can also influence the immune response in melanoma, such as increased local infiltration of NK cells in melanoma after inhibition of Beclin-1, ATG5 or p62 correlated with increased expression of CCL5 and improved prognosis. Loss of BNIP3 decreased macrophage phagocytosis of dying melanoma cells. Besides, autophagy activity is associated with MDSC- mediated suppression of anti-melanoma immunity, melanoma adaption to fluctuating O_2_ or pH, and outcome of targeted therapy with temozolomide and sorafenib

Autophagy is a cellular catabolic mechanism wherein proteins, bulk cytoplasm, and/or organelles are sequestered inside double-membrane intracellular vesicles for subsequent recycling via lysosomes (Fig. 1). Therefore, autophagy plays a crucial role in maintaining cellular homeostasis via the elimination of unfolded proteins and damaged organelles. Autophagy has often been considered a system that suppresses tumor development during the initial stage of carcinogenesis. However, the advancement of cancer, including melanoma, has been linked to the tumor-promoting function of autophagy. Autophagy-related 5 (ATG5) downregulation promotes the proliferation of *BRAF*^*V600E*^ melanocytes, supporting the suppressive role of autophagy in tumorigenesis [50]. In contrast, enhanced basal autophagy in melanoma cells carrying the *BRAF*^*V600E*^ mutation, causing chronic ER stress, is related to chemoresistance, which can be reversed by chemical chaperones (4-phenylbutyric acid), accompanied by decreased basal autophagy and increased susceptibility to cell death induction [51]. One reason for the tumor-promoting role of autophagy is that active autophagy is an adaptive mechanism to microenvironment insults for melanoma cells. Therefore, inhibition of autophagy makes melanoma cells vulnerable to fluctuating oxygen pressure [52] and low pH [53], both of which are associated with tumor progression and tumor metastasis. Furthermore, autophagy may be a mechanism through which melanoma cells mitigate the effects of drug activity and drug-induced alterations in the TME. Therefore, the autophagy machinery exhibits a significant association with clinical outcomes and plays a substantial role in the development of drug resistance. In a phase II trial of temozolomide (an oral alkylating agent) and sorafenib (an oral multikinase inhibitor), patients with melanoma displaying high autophagy activity had a worse clinical outcome [54]. Conversely, autophagy inhibition with either hydroxychloroquine or inducible shRNA against ATG5 resulted in significantly augmented temozolomide-induced cell death in aggressive melanoma spheroids [55]. Another example is that the efficacy of vemurafenib (a BRAF inhibitor) is enhanced by the ectopic expression of miR-216b to attenuate autophagy both in vitro and in vivo [56]. In addition, natural products are attractive sources of molecules that effectively kill melanoma cells. For example, 7-hydroxydehydronuciferine, a dehydroaporphine that is isolated from the leaves of *Nelumbo nucifera* Gaertn cv. *Rosa-plena*, induces cytotoxicity through apoptosis- and autophagy-dependent cell death in the context of melanoma both in vitro and in vivo [57].

Because of the relatively high responsiveness of melanoma to immunotherapy, studies have focused on the relationship among autophagy, melanoma and the immune response, revealing that the role of autophagy is dynamic and multifaceted and induces different effects in different scenarios. The loss of BCL2-interacting protein 3 (BNIP3, an inducer of autophagy) in melanoma cells did not alter apoptosis induction but attenuated the phagocytosis-mediated clearance of dying melanoma cells [58]. Forced expression of microtubule-associated protein 1 light chain 3 beta (*MAP1LC3B*, encoding an autophagy initiator) restored the susceptibility of phosphatase and tensin homolog (PTEN)-deficient melanoma cells to T-cell-mediated cell killing [59]. The mouse model showed that the T-cell-mediated response was the same for melanoma cells with functional autophagy machinery, melanoma cells without genes related to autophagy, and melanoma in which autophagy is blocked by chloroquine [60]. In contrast to the neutral or positive role on T-cell and phagocytic immunity, increased local infiltration of natural killer (NK) cells after inhibition of Beclin-1, ATG5 or p62/Sequestosome 1 (SQSTM1) in melanoma cells correlated with increased expression of the chemokine C–C motif chemokine ligand 5 (CCL5) has been reported. High expression of CCL5 has also been correlated with prolonged patient survival [61, 62]. Finally, inhibiting autophagy in myeloid-derived suppressor cells (MDSCs), a group of immune cells that accumulate in tumors to dampen the immune reaction, shows the potential to slow melanoma expansion and trigger strong antimelanoma immune reactions in mice [63].

### Pyroptosis

**Fig. 2:**
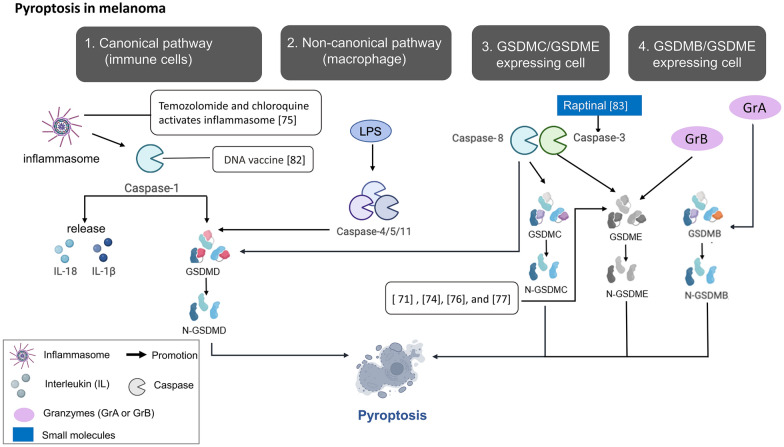
Pyroptosis and its role in melanoma. Pyroptosis can be activated by four pathways: (1) the canonical pathway with inflammasome assembly and caspase-1 activation (2) the noncanonical pathway with caspase-4/5/11 activation by direct binding of its N-terminal CARD domain to intracellular LPS. (3) Caspase 3/8-mediated pathway with GSDMC or GSDME cleavage (4) Granzyme-mediated pathway with granzyme A and B, secreted from cytotoxic T lymphocytes and NK cells respectively, can cleave GSDMB and GSDME, respectively. Research of pyroptosis on melanoma was expressed in white squares with reference number in parenthesis. The exogenous molecules are marked in blue squares especially. GSDME cleavage by activated caspase 3 has been observed by chemotherapeutic drugs, BRAF and MEK inhibitors/ PDPK1 and MEK inhibitors, and ROS production with iron dextran or raptinal, a caspase 3 activator. Caspase-1 DNA was ever included in anticancer DNA vaccine to induce pyroptosis of melanoma cells. Pyroptosis can also be induced by temozolomide and chloroquine via autophagy inhibition and inflammasome activation

Pyroptosis is a lytic programmed cell death characterized by pore formation in the plasma membrane via the oligomerization of cleaved gasdermin. It was first discovered in 1992 in macrophages infected with the gram-negative bacterial pathogen *Shigella flexneri* [64], and the term pyroptosis was coined in 2001 [65]. Initially, pyroptosis was thought to involve only the death of monocytes caused by caspase-1 activation [66]. However, research into pyroptosis has increased, revealing a wide range of triggering conditions, such as cancer. This expansion in pyroptosis-related environments coincided with the identification of the gasdermin family. The gasdermin superfamily includes gasdermin A/B/C/D (GSDMA/B/C/D), gasdermin E (GSDME, also known as DFNA5), and DFNB59 (Pejvakin, PJVK) in the context of human biology [67]. Except for Pejvakin, these proteins share a common structural arrangement featuring two conserved segments: the pore-forming domain in the N-terminus and the repressor domain in the C-terminus. Theoretically, gasdermins form pores after the N-terminal domain is dissociated from the C-terminal domain via caspase or granzyme cleavage. Among these gasdermins, GSDMD and GSDME are the most intensively studied in the context of pyroptosis. Generally, pyroptosis can be initiated through one of the following four routes: (1) the canonical pathway: inflammasome assembly triggers caspase-1 activation, leading to GSDMD cleavage and subsequent release of IL-1β and IL-18; (2) the noncanonical pathway: caspase-4/5/11 can be activated through direct binding of the N-terminal caspase-activation and recruitment domain (CARD) to intracellular lipopolysaccharide (LPS) [68] with subsequently activated caspase-4/5/11 cleaving GSDMD to release N-GSDMD, which forms pores on the cell plasma membrane; (3) the caspase 3/8-mediated pathway: previously, caspases 8 and 3 were thought to be initiator and effector caspases, respectively, in the apoptosis pathway; however, chemotherapeutic drugs have been shown to induce caspase-3-mediated pyroptosis [69], and GSDMD can be cleaved by caspase 8 to execute pyroptosis, while caspase 8 autoprocessing is a feasible possibility [70]; and (4) the granzyme-mediated pathway: granzymes A and B, which are released by NK cells and cytotoxic T lymphocytes, respectively, cleave GSDMB and GSDME to initiate pyroptosis (Fig. 2). In the caspase 3-mediated noncanonical pyroptosis pathway activated by chemotherapeutic agents, DNA-binding and DNA-modifying drugs such as doxorubicin, cisplatin, and actinomycin-D, along with topoisomerase inhibitors such as topotecan, CPT-11, etoposide, and mitoxantrone, induce pyroptosis in GSDME-positive cancer cells such as SH-SY5Y neuroblastoma and MeWo skin melanoma cells [71]. GSDME expression can lead to a switch from tumor necrosis factor (TNF)-induced apoptosis to pyroptosis mediated through caspase 3 activity [71]. Antibiotic chemotherapy, including daunorubicin, doxorubicin, epirubicin, and actinomycin-D, was also reported to increase the nuclear translocation of PD-L1, promote GSDMC expression and activate caspase-8. Activated caspase 8 further cleaved GSDMC and induced pyroptosis in breast cancer cells [69]. In addition to inhibiting antitumor immunogenicity to enable PD-L1 to bind PD-1 on T lymphocytes, PD-L1 on melanoma cells trans-interacts with PD-1 on melanoma cells, leading to cell proliferation and in vivo tumor growth [72]. Pyroptosis has been reported to be triggered by nuclear PD-L1 under hypoxic conditions and has been observed in various cancer cell types, including breast, liver, lung, and ovarian cancers and melanoma [69]. Moreover, the GSDME protein levels in cancer cells are not as invariable as those in normal cells [73], and BRAF/MEK inhibitors can promote the cleavage of GSDME and the release of high-mobility group box 1 (HMGB1), which are markers of pyroptotic cell death in melanoma cells [74]. After the development of resistance to BRAF/MEK inhibitors, these resistant melanoma cell lines are susceptible to pyroptosis, which can be induced by etoposide or doxorubicin [74], and the combination of temozolomide and chloroquine [75]. The combined use of a phosphoinositide-dependent kinase 1 (PDPK1) inhibitor (GSK2334470) and a MEK inhibitor (trametinib) suppressed NRAS-mutant xenograft growth and induced GSDME-associated pyroptosis in NRAS-mutant melanoma model mice [76]. In addition, reactive oxygen species (ROS) induce pyroptosis via BAX (BCL2 associated X) recruitment to release cytochrome c and subsequent caspase-3 activation. In contrast to the lack of an effect mediated by iron supplementation on tumor growth reduction in *GSDME*-knockdown mice, iron-induced ROS production via iron dextran decreased melanoma growth in *GSDME*^wt^ mice, demonstrating that ROS-induced pyroptosis is GSDME dependent [77].

Since pyroptosis is a form of inflammatory programmed necrosis, the role of pyroptosis in the TME has been studied. After treatment with BRAF/MEK inhibitors, melanoma cells lacking GSDME exhibited impaired infiltration of tumor-associated T cells, the number of activated dendritic cells was diminished, and a higher incidence of tumor regrowth after cessation of drug treatment was recorded [74]. Pyroptosis-associated genes were utilized in modeling aimed at forecasting melanoma prognosis, predicting immunotherapy responses, and discerning immune microenvironment attributes [78], with applicability extending to metastatic melanoma as well [79]. The increased expression of pyroptosis-related genes correlated with increased infiltration of tumor-associated B cells, plasma cells, CD8^+^ T cells, activated memory CD4^+^ T cells, regulatory T cells (Tregs), and M1 macrophages, while the levels of resting NK cells, M2 macrophages, M0 macrophages, and resting mast cells were reduced. This pattern of gene expression and cell contents corresponded to a more favorable prognosis [78]. Nevertheless, pyroptosis might exhibit tumor-promoting characteristics stemming from inflammasome activation in the context of chronic inflammation. This possibility was supported by the finding of a significantly reduced incidence of lung cancer and decreased lung cancer mortality in a trial after the activity of the major product of inflammasome activation, IL‐1β, was inhibited by a specific antibody, canakinumab [80]. A recently proposed hypothesis suggests that the results hinge on whether inflammasome activation occurs predominantly within the tumor or within immune cells. Persistent inflammasome activation within the TME leads to tumor promotion and immune suppression. In contrast, when inflammasomes are activated within the immune system, specifically within dendritic cells, they contribute to attenuated melanoma progression [81]. In addition, active caspase 1, a genetic adjuvant in DNA vaccination against cancers, promoted pyroptosis to kill melanoma cells in mice [82]. In addition, the caspase-3 direct activator raptinal, a bifluorene–dicarbaldehyde compound, induced pyroptosis in both human and mouse melanoma cell line models and delayed tumor growth in vivo; this study suggests that the release of damage-associated molecular patterns (DAMPs) and inflammatory cytokines is dependent on caspase activity and GSDME expression [83]. Therefore, pyroptosis induction may be a strategy to treat melanoma, but to determine how to manipulate pyroptosis to eliminate its tumor suppression effect, more study is needed.

### Necroptosis

**Fig. 3:**
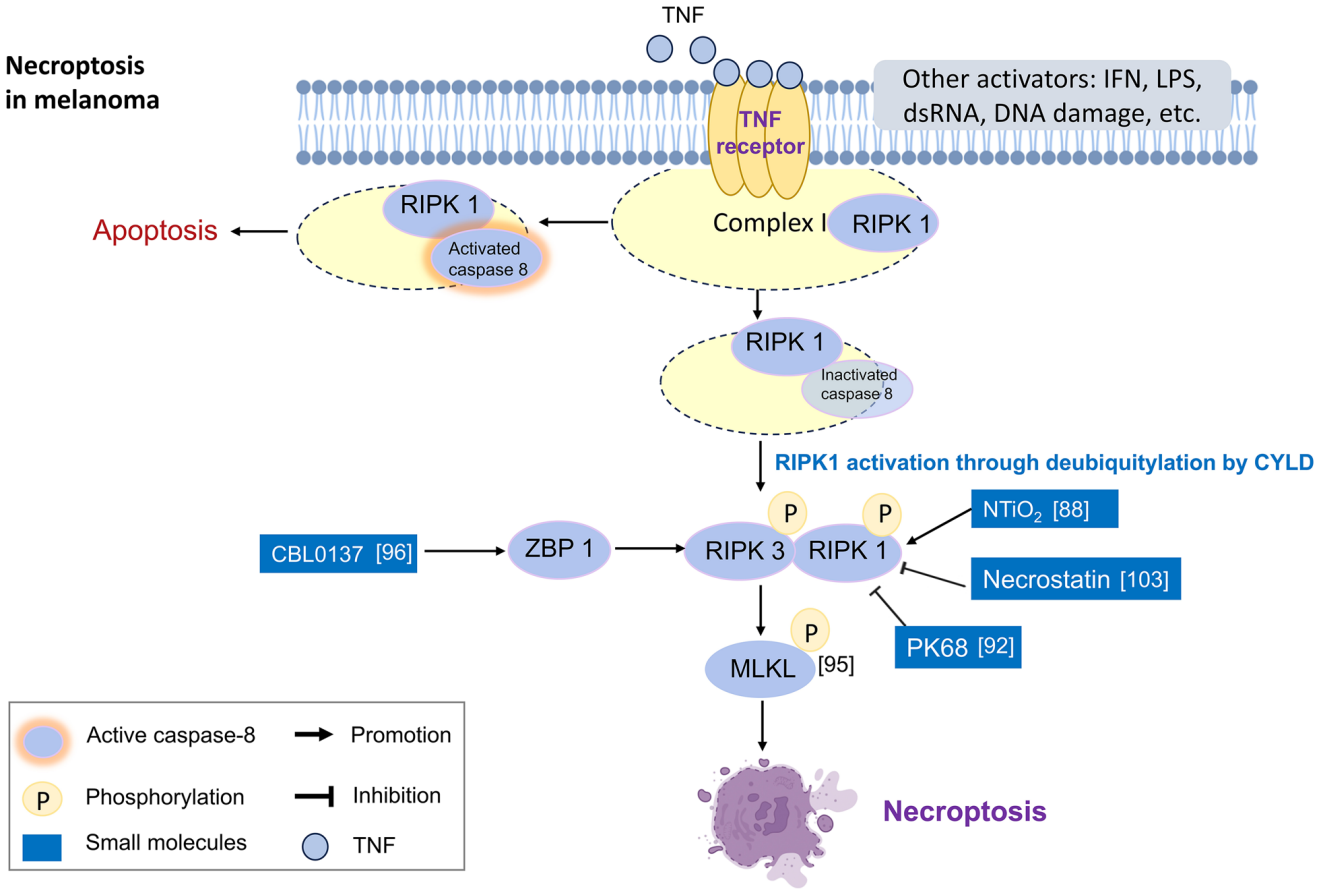
Schematic diagram of necroptosis and its effects on melanoma. Necroptosis can be triggered by activation of death receptors or Toll-like receptors. Through interaction of activated RIPK1 with RIPK3, and subsequently, RIPK3 phosphorylates MLKL. Pore formation on cell membrane is the final step of necroptosis. Research of necroptosis on melanoma was expressed in white squares with reference number in parenthesis. The exogenous molecules are marked in blue squares especially. While unsure effect of RIPK1 inhibition on melanoma metastasis, direct intratumor delivery of MLKL mRNA can inhibit melanoma tumor growth and metastasis and augment the efficiency of immune blockade therapy. Besides, NTiO_2_, BAY 87–2243 and CBL0137 are the agents with the ability to induce necroptosis in melanoma cells

Necroptosis is a caspase-independent cell death pathway recognized as programmed necrosis. Furthermore, necroptosis can be triggered by the substantial production of ROS, hyperactivation of poly (ADP-ribose) polymerase 1 (PARP1) and depletion of ATP [84, 85]. Through activating death receptors, Toll-like receptors or cytosolic nucleic acid sensors that induce type I interferon (IFN-I) and TNFα production in an autocrine feedback loop, necroptosis can be triggered. Initially, receptor-interacting protein kinase 1 (RIPK1) is deubiquitylated by CYLD lysine 63 deubiquitinase (CYLD) and can recruit receptor-interacting protein kinase 3 (RIPK3). Then, the RIPK1/RIPK3 complex recruits and phosphorylates mixed lineage kinase domain-like pseudokinase (MLKL). Finally, phosphorylated MLKL oligomerizes and forms a large pore on the plasma membrane, leading to necroptotic cell death [86] (Fig. 3). Crosstalk between the extrinsic pathway of apoptosis and necroptosis is mediated through the activation of death receptors, and necroptosis can be activated when the intracellular apoptosis signaling pathway is inhibited [87]. A shift from the autophagic flux response to necroptotic cell death has been observed when nitrogen-doped titanium dioxide (N-TiO_2_) nanoparticles were photoactivated in melanoma cells [88]. Several investigations have reported that the potential to trigger necroptosis in melanoma might be inhibited due to low expression levels of both CYLD [89] and RIPK3 in melanoma cell lines [90, 91].

With the manipulation of upstream RIPK1 and the effector protein of MLKL in the necroptosis pathway, the relevance of necroptosis to melanoma progression and metastasis is not clear. For example, a novel RIPK1 inhibitor, PK68, significantly suppressed the lung metastasis of melanoma cells in mice [92] The inhibition of RIPK1 in mice by knock-in inactivated RIPK1 D138N or the murine-potent inhibitor GNE684 showed no effect in reducing lung metastases of B16 melanoma cells, although it mitigated collagen antibody-induced arthritis, skin inflammation caused by mutation of Shank-associated RH domain interactor (SHARPIN, which is associated with NF-kappa-B activation and regulation of inflammation), or colitis caused by deletion of Nemo (also known as IKK-γ. a subunit of activated NF-κB) [93]. However, direct intramelanoma delivery of either the RIPK3 gene via adenovirus or mRNA encoding MLKL, a necroptosis executioner, elicited both necroptosis and potent antitumor immunity in melanoma model mice [94, 95]. The combination of MLKL mRNA and anti-PD1 treatment revealed better antitumor activity compared with anti-PD-1 alone, and the effect depended on CD4 and CD8 T cells under the control of type I interferon signaling and basic leucine zipper transcription factor ATF-like 3 (Batf3)-dependent dendritic cells [95]. Anti-PD-1 unresponsiveness in mouse models of melanoma was reversed by a small molecule, curaxin CBL0137, which potently activated Z-form nucleic acid binding protein 1 (ZBP1)-dependent necroptosis by triggering Z-DNA formation in tumor-infiltrating fibroblasts, irrespective of the potential for low necroptotic gene expression in melanoma cells [96]. In addition, necroptosis and ferroptosis may be the mechanisms underlying melanoma cell death simultaneously; for example, BAY 87-2243-induced melanoma cell death due to ROS accumulation through mitochondrial complex I inhibition was attenuated by necrostatin (a necroptosis inhibitor), knockdown of RIPK1 or MLKL, ferrostatin (a ferroptosis inhibitor) or knockdown of GPX4 but not treatment with the pancaspase inhibitor z-VAD-FMK [97].

### Ferroptosis

**Fig. 4:**
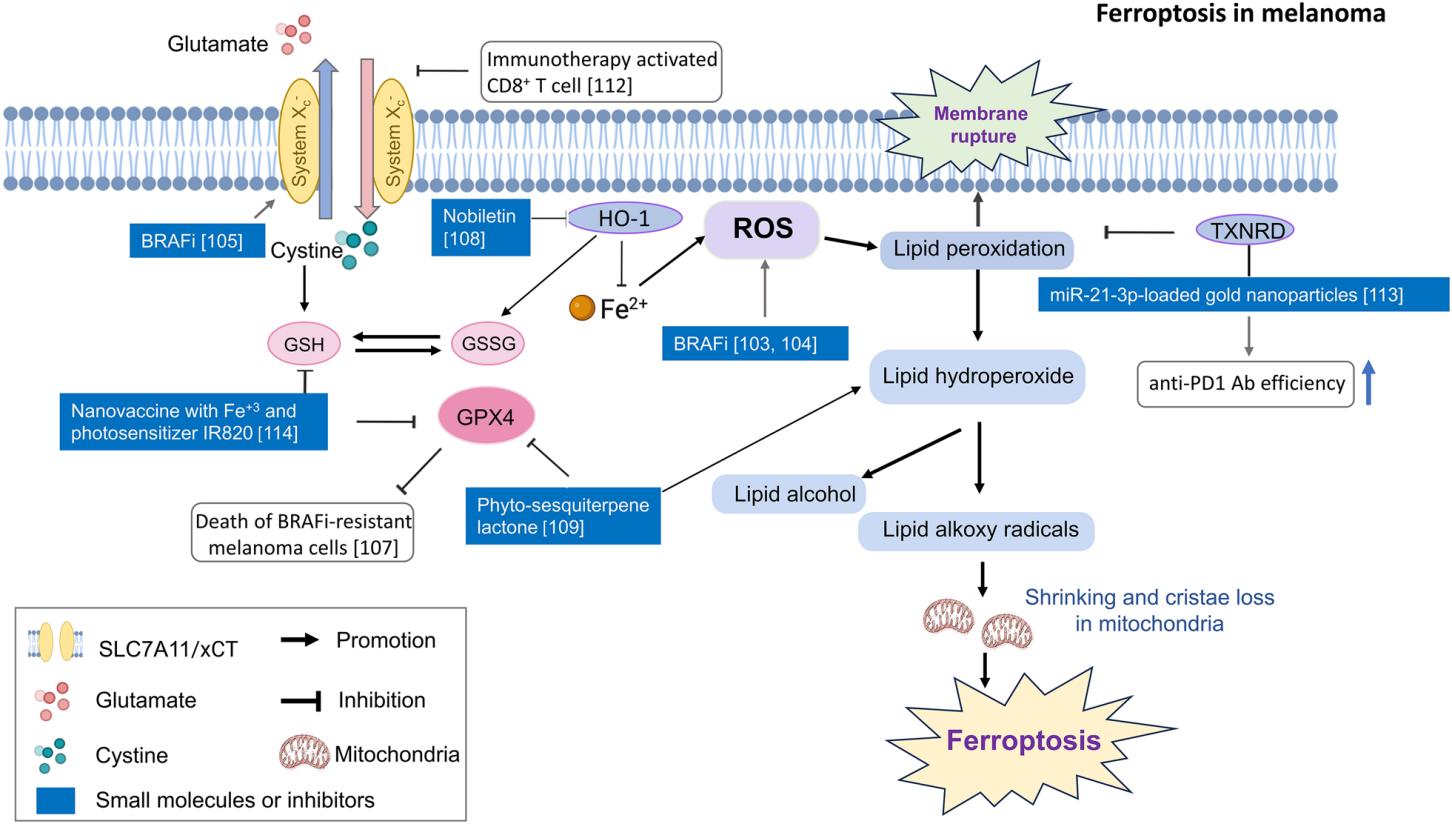
The relationship between ferroptosis and melanoma. Ferroptosis is characterized by the overwhelming production of ROS and accumulation of iron-dependent lipid peroxides. Research of ferroptosis on melnoma was expressed in white squares with reference number in parenthesis. The exogenous molecules are marked in blue squares especially. Ferroptosis can be induced in melanoma cells by GPX4 inhibition in the situation of BRAF inhibitor resistance or with the application of plant-derived phyto-sesquiterpene lactone. Nobiletin can also induce ferroptosis in melanoma cells through another pathway of GSK3β-mediated Keap1/Nrf2/HO-1 signaling. In addition, IFNγ release after immunotherapy can drive ferroptosis by downregulating the expression of system Xc^−^. which can be further enhanced by the delivery of miR-21-3p-loaded gold nanoparticles

Ferroptosis is an orchestrated caspase-independent mechanism underlying cell death distinguished by the excessive generation of ROS and accumulation of iron-associated lipid peroxides [98–100]. Free radical attack on polyunsaturated fatty acids in the membrane results in the formation of lipid hydroperoxides (L-OOH), which can be converted into highly reactive lipid alkoxy radicals (L-O•) by ferrous ions and thus induce the initiation of ferroptosis. L-OOH can be reduced to lipid alcohol (L-OH) by selenoprotein glutathione peroxidase 4 (GPX4) in the presence of glutathione (GSH), an undependable hydrophilic cellular antioxidant that prevents lipid peroxidation. Therefore, ferroptosis can be triggered by inhibition of GSH biosynthesis or inhibition of GPX4 (Fig. 4). System X_c_^−^, a transporter responsible for exchanging cysteine (Cys) and glutamic acid (Glu), comprises the catalytic subunit xCT (also known as solute carrier family 7 member 11, SLC7A11), which is the light chain, and the regulatory subunit 4F2hc (also known as solute carrier family 3 member 2, SLC3A2), which is the heavy chain. These subunits are interconnected by disulfide bonds, forming a pivotal upstream hub within the System Xc − /GSH/GPX4 pathway. This pathway relies on the System X_c_^−^ − mediated import of cysteine, which is utilized for the biosynthesis of glutathione (GSH). Therefore, erastin, which interferes with system X_c_^−^, is a ferroptosis inducer.

Cancer cells are reported to be highly vulnerable to disruptions to thiol metabolism and an overabundance of iron [101]. In melanoma, BRAF inhibitors can sensitize melanoma cells to agents that cause ferroptosis, which reduces the abundance of SLC7A11 transcripts [102]. The sensitivity of melanoma cells to ferroptosis relies on their enhanced dependence on oxidative phosphorylation (OXPHOS), which is upregulated by BRAF inhibitors in BRAF-mutant melanoma cells, resulting in the accumulation of ROS [103, 104]. With the development of resistance to BRAF inhibitors, ferroptosis plays a role in melanoma cell viability. Using a mouse model and melanoma cell lines generated from mouse tumors treated with vemurafenib (BRAF inhibitor) or not, it has been shown that the acquisition of drug resistance is associated with an increase in mitochondrial OXPHOS, growth dependency on the glutamine supply and a compensated increase in glutathione levels, which is associated with strong activation of the nuclear factor erythroid 2-related factor-2 (NRF2) pathway and increased xCT expression [105]. The increase in the GSH level and increased xCT expression indicated that these melanoma cells may be more resistant to ferroptosis induction. In contrast, by clustering gene expression in 53 human melanoma cell lines, melanoma dedifferentiation was linked to resistance to MAPK inhibitors and immunotherapy, lower basal levels of GSH and increased sensitivity to ferroptosis [106]. The basal GSH levels were lower in vemurafenib-resistant cell lines than in matched drug-naïve cells [106]. Moreover, loss of *GPX4* induced the death of BRAF-mutant therapy-resistant cells via ferroptosis in vitro and prevented tumor relapse after treatment in vivo [107]. Although the basal GSH level in MAPK inhibitor-resistant melanoma cells was not conclusive by Khamari et al*.* [105] or Tsoi et al*.* [106], all their studies revealed the need for GSH level increases for melanoma cells to overcome ferroptosis, and the induction of ferroptosis may be a therapeutic target, even in MAPK inhibitor-resistant melanoma cells. In addition, nobiletin, isolated from citrus peel, induced ferroptosis in human skin melanoma cells through the GSK3β-mediated Keap1/Nrf2/HO-1 signaling pathway [108]. The phyto-sesquiterpene lactone DET and its derivative DETD-35 cause lipid ROS to accumulate, which leads to ferroptotic cell death in both BRAF-sensitive and BRAF-resistant V600E melanoma cells [109]. These studies show that natural products or extracts from plants are possible treatment alternatives to MAPK inhibitors.

In contrast to alternative forms of cellular demise, such as apoptosis, pyroptosis, and necroptosis, the factors of which are clearly recognized by the immune system [110], whether inducing or instigating ferroptosis through external or internal mechanisms can lead to a similar "physiological function" as these other forms of RCD is unclear. Notably, GPX4 can abrogate lipoxygenase and cyclooxygenase function by lowering lipid peroxide levels [111]. It seems likely that GPX4 activity can exert a large effect on the release of both proinflammatory and anti-inflammatory lipids [110] when GPX4 activity is hampered. A previous investigation demonstrated that CD8^+^ T cells activated by immunotherapy released IFNγ, causing a reduction in the expression of SLC3A2 and SLC7A11, subsequently leading to high lipid peroxidation specific to ferroptosis. As a result, ferroptosis is induced within tumor cells [112]. Systemic delivery of miR-21-3p-loaded gold nanoparticles increased the efficacy of anti-PD-1 antibodies by promoting ferroptosis in preclinical melanoma model mice [113]. Administration of a biomineralized nanovaccine containing Fe^3+^ and the photosensitizer IR820 through intratumoral injection triggered ferroptosis and localized immunogenic cell death. This approach enhanced the effectiveness of CTLA-4 blockade therapy in model mice [114]. In addition, a model established with ferroptosis-related genes or long noncoding RNAs (lncRNAs) was generated to predict the prognosis of melanoma, showing the correlation of increased local immune cell infiltration with an increased response to immunotherapy [115, 116]. All these studies suggest that the combination of ferroptosis induction-based therapy and immunotherapy is a potential option for treating melanoma.

### Cuproptosis

**Fig. 5:**
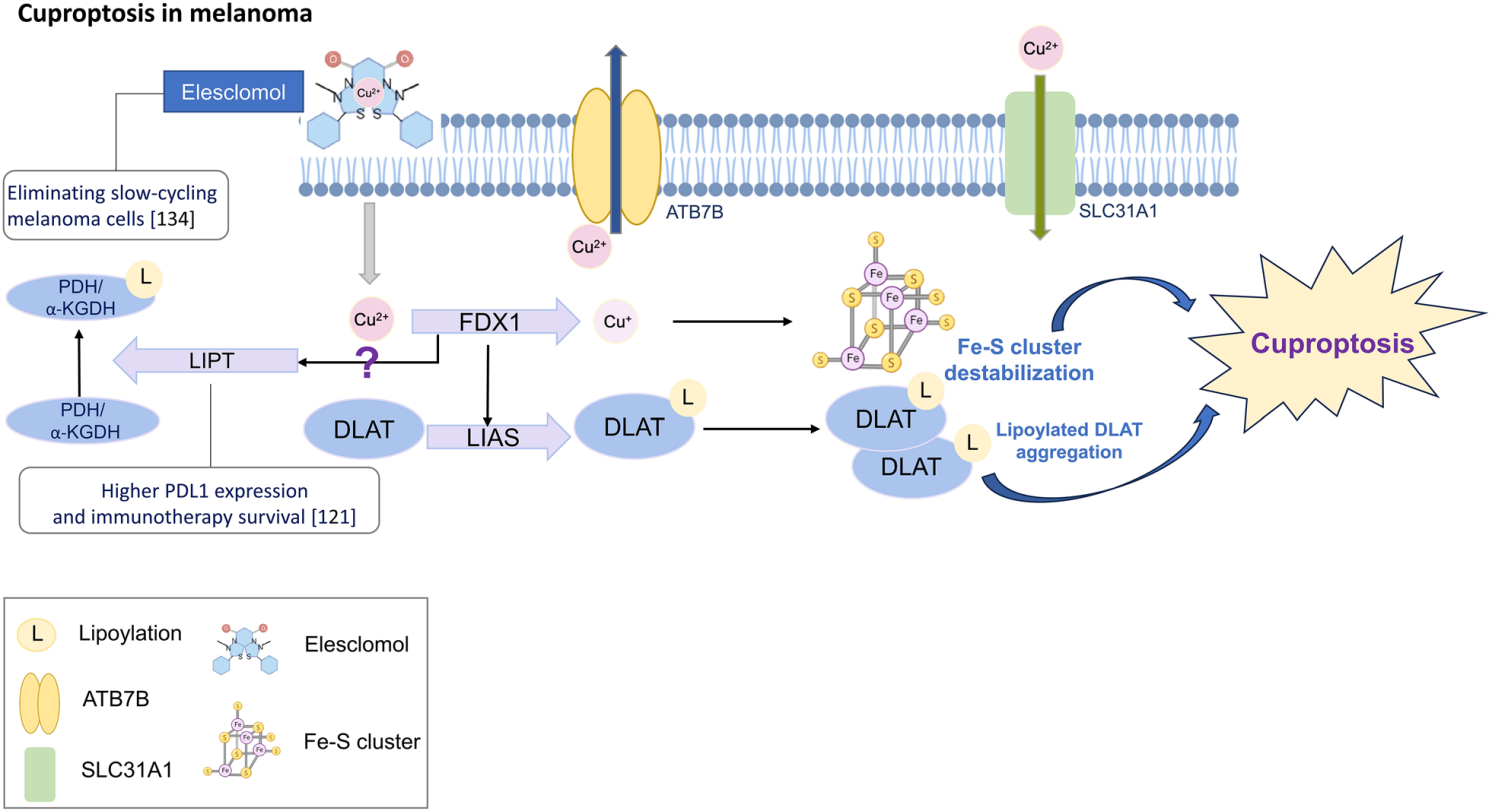
Schematic diagram of cuproptosis and correlation between cuproptosis and melanoma. Copper ions can cross the cell membrane into the intracellular space with a copper ionophore, and their concentration can be regulated by copper importers/exporters (SLC31A1/ATP7A and B). FDX1 not only reduces divalent copper to more toxic monovalent copper but also regulates protein lipoylation. Intracellular copper can bind lipoylated proteins directly, leading to their aggregation and further occurrence of cuproptosis. Copper ions also contribute to Fe-S cluster loss, which is another mechanism of cuproptosis. Related researches are expressed in white squares with reference number in parenthesis. Elesclomol, a copper ionophore, can induce cuproptosis, and was shown to eliminate slow-cycling melanoma cells. A higher expression level of LIPT1, responsible for transferring lipoic acid to the E2 subunits of AKGDH and pyruvate dehydrogenase PDH, is related to longer survival after immunotherapy

Cuproptosis, a recently discovered RCD modality, was first described in a 2022 publication by Tsvetkov et al*.* [117]. This distinct mode of cellular demise is activated by the accumulation of intracellular copper, which results in the clustering of mitochondrial lipoylated proteins and the disruption of Fe-S cluster proteins. In contrast to other types of controlled cell death, such as apoptosis, necroptosis, and ferroptosis, inhibitors such as ferrostatin-1 and necrostatin-1 and antioxidants such as N-acetyl cysteine exert no effect on cuproptosis. In addition, cuproptosis functions through the mitochondrial respiration chain complex, not through ATP production, as demonstrated by the fact that treatment with copper ionophores did not significantly reduce basal or ATP-linked respiration. In the study by Tsvetkov et al*.*, intracellular copper shuttled by elesclomol, a copper ionophore, directly bound to lipoylated mitochondrial proteins, leading to their aggregation and subsequent loss of Fe-S cluster proteins. This proteotoxic stress condition ultimately resulted in cuproptosis (Fig. 5). The lipoylated mitochondrial proteins identified, including DBT, GCSH, DLST, and DLAT, were involved in regulating carbon entry points to the tricarboxylic acid (TCA) cycle [118, 119].

Ferredoxin 1 (FDX1), a reductase that converts Cu^2+^ to its toxic form, Cu^1+^, and a direct binding target of elesclomol [120], was established as the upstream positive regulator of DLAT lipoylation, and it was essential for copper binding. Furthermore, mass spectrometry analysis indicated that copper ionophore treatment led to the loss of Fe–S cluster proteins in an FDX1-dependent manner.

The discovery of cuproptosis has sparked numerous studies aimed at understanding its role in tumor development and prognosis, particularly in melanoma. Through July 2023, 10 articles discussing cuproptosis and melanoma had been published on PubMed [121–130]. Eight of these studies described the correlation between cuproptosis-related genes (CRGs) and melanoma prognosis [121–123, 126–130], and the other two studies presented a discussion of the impact of cuproptosis-related lncRNAs [124, 125]. CRGs were assessed in cutaneous melanoma samples in five studies [121, 126–128, 130] and uveal melanoma in two studies [123, 129]. In addition to prognosis prediction, the expression of CRGs was related to the regulation of the TME with differential immune cell infiltration [121–123, 126–130], response to immunotherapy [126, 130], and different chemotherapeutic and targeted drug sensitivities [122, 128, 130].

Based on the work of Tsvetkov et al*.*, the CRGs include FDX1, LIPT, LIPT1, DLD, DLAT, PDHA1, PDHB, MTF1, GLS, CDKN2A, SLC31A1, ATP7A, and ATP7B [117]. These CRGs not only serve as prognostic indicators but are also used to identify intersecting genes, such as AIM2, LAG, SLC39A6, TMEM117, PTPRC, and KIF14, for building prognostic models [122]. Among these genes, LIPT1 has emerged as a advantageous prognostic marker for individuals with melanoma [121]. Additionally, the expression of the *LIPT1* gene has been linked to PD-L1 expression, regulatory T-cell infiltration, and longer survival in melanoma patients who were treated with immunotherapy [121]. Moreover, CRGs have been used to identify pyroptosis-related lncRNAs, leading to the establishment of prognostic models [124, 125]. The immune microenvironment landscape correlated with these lncRNA prognostic models, revealing significant differences in regulatory T-cell infiltration rates and better immunotherapy responses in lower-risk groups [124, 125].

Although the role of cuproptosis in melanoma progression is attractive, the exact mechanism is not known by CRG analysis, and the upstream regulators and downstream effectors remain unclear. In-depth studies are needed to decipher the mechanisms underlying cuproptosis-related proteins, their interactions with other RCD pathways, and the potential therapeutic applications of manipulating cuproptosis as a target. For example, FDX1 has been identified as a positive upstream regulator of cuproptosis, and its expression is decreased in high-risk melanoma, as classified by CRGs; however, its knockdown inhibited melanoma cell proliferation in vitro [122]. Similarly, before the discovery of cuproptosis, copper was noted to cause cell death in the 1980s [131]. Copper ionophores were utilized to treat cancer, and the cytotoxicity was inferred to be ROS mediated at that time [132]. Elesclomol was shown to transport copper to the mitochondria to induce oxidative stress in melanoma cells in a 2012 study [133]. Elesclomol can selectively kill slow-cycling melanoma cells, which are considered multidrug resistant [134]. However, the clinical use of elesclomol combined with paclitaxel yielded mixed results in clinical trials [135, 136]. Clinical trials investigating disulfiram (a copper ionophore) to treat melanoma resulted in unsatisfactory data and were unpublished [137]. The mitochondrial morphological change remains to be determined during cuproptosis [138]. In addition, another copper ionophore, thiomaltol induced rapid lysosomal accumulation of copper, concurrent with the onset of apoptosis. This finding implied a mechanism other than cuproptosis and ROS accumulation to explain copper cytotoxicity [139]. In fact, there was one study stating that copper cytotoxicity was associated with the disturbance of proteostasis due to protein misfolding and aggregation by the interaction of copper with proteins [140]. GSH can protect cells from preventing copper ion interactions with proteins [140]. Regardless of cuproptosis, copper-induced oxidative stress or protein misfolding and aggregation, copper can form a cogroup of many enzymes and proteins that plays a crucial role in maintaining mitochondrial homeostasis [138]. All these findings necessitate further investigation of the mechanism regulating cuproptosis, especially the impact on mitochondrial dysfunction, and its possible role in tumor metabolic pathway switching.

## Common mechanisms among these nonapoptotic RCDs

Although pyroptosis, ferroptosis, necroptosis, and cuproptosis present distinct biological processes with unique morphological features and triggers (Table 2), there are certain commonalities among them. For example, the caspase family not only functions in apoptosis but also regulates other types of RCDs, depending on the cellular context and stimuli. Pyroptosis can be triggered by caspase 1 in the canonical pathway, caspase 4/5/11 in the noncanonical pathway, or caspase 3/8 in GSDMC- or GSDME-expressing tumor cells, including melanoma cells. Although necroptosis is a caspase-independent RCD, its activation depends on caspase 8 inactivation. Similarly, the execution of autophagy-dependent cell death is caspase independent, but caspases can modulate it, such as the induction of autophagy-dependent cell death in fibroblasts and monocytoid cells by caspase 8 inhibition, which requires ATG7 and beclin-1 [141]. Ferroptosis and cuproptosis are both caspase-independent RCDs. In addition to caspase, ROS are another important modulator that induces these RCDs. ROS caused by iron supply can lead to pyroptosis of melanoma cells by GSDME cleavage [77]. The accumulation of ROS induces necroptosis and can form positive feedback [142]. The occurrence of ferroptosis depends on lipid peroxidation generated by ROS attack. Although the cell death mechanism is not related to ROS in cuproptosis, copper can induce cell death through an increase in ROS[143]. Since mitochondria are the main source of ROS, the generation of these RCDs is often associated with mitochondrial dysfunction.

**Table 2:** Morphological features and known triggers of autophagy dependent cell death, pyroptosis, necroptosis, ferroptosis and cuproptosis

Cell death	Autophagy dependent cell death	Pyroptosis	Necroptosis	Ferroptosis	Cuproptosis
*Features or triggers*					
Morphological features	Formation of intracellular vesicles	Cell swelling; rupture of plasma membrane; moderate chromatin condensation	Rupture of plasma membrane; pore formation	Shrinking mitochondria with decreased crista; larger membrane rupture	Not identified
Triggers	Excess activation of autophagy (a normal catabolic mechanism)	Damage-associated molecular pattern (DAMP); pathogen associated molecular pattern (PAMP), cytosolic lipopolysaccharide, activation of caspase 3/8	Activation of death receptors, Toll-like receptors or cytosolic nucleic acid sensors	ROS accumulation and lipid peroxidation	Copper accumulation by copper inotropes

*BRAF*-mutant melanoma cells can become resistant to BRAF/MEK inhibitors after long-term exposure. However, these resistant cells can be manipulated to be sensitive to the induction of pyroptosis and ferroptosis [74, 107, 109]. Inhibition of autophagy activity can increase the efficiency of BRAF inhibitors [56]. The performance of necroptosis or cuproptosis in BRAF inhibitor-resistant melanoma cells is not well evidenced. Apart from targeted therapy, the efficiency of immunotherapy in melanoma has been shown to be enhanced by the induction of necroptosis or ferroptosis [95, 113]. Inhibition of autophagy processing, expression of pyroptosis- or cuproptosis-related genes also showed an impact on the TME and local immunity [59, 62, 78, 121–123, 126–130]. These RCDs can be triggered at the same time, such as ferroptosis and necroptosis induction by BAY-2743 [97]. Ferroptosis inducers sorafenib and erastin can also enhance cuproptosis in primary liver cancer cells by increasing copper-dependent lipoylated protein aggregation [144]. All these findings support the possible interchangeability of these RCDs on the dying pathway of melanoma cells, and what we know is not enough.

## Conclusions and perspectives

Effective treatment for metastatic melanoma has been historically challenging, with a 5-year survival rate of only 15–20% recorded between 1992 and 2011 [145]. However, with the introduction of targeted therapy and immunotherapy in the 2010s, the 3-year survival rate increased to 39.4% [2]. Despite these advancements, the continuously increasing incidence of melanoma highlights the ongoing need for more effective therapeutic approaches.

In the first part of this review, we provide an overview of the molecular-level pathogenesis of melanoma and describe current treatment options, focusing on systemic therapy involving targeted therapy and immune blocker therapy for advanced melanoma. Although significant progress has been made, certain limitations persist, such as poor response rates to immune checkpoint blockers and targeted therapy in patients with subtypes such as acral, mucosal, and uveal melanoma. Additionally, secondary treatment resistance and the need for reliable biomarkers to guide treatment selection remain important challenges.

The application of immunotherapy and targeted therapy has ushered in a new era of melanoma treatment. However, these approaches alone are not sufficient due to treatment resistance and loss of response. Synergistic therapy, combining different treatment modalities, holds great potential and may become the future mainstay of cancer therapy. As evasion from RCD is a key feature of tumorigenesis, targeting various nonapoptotic RCDs with pharmacological small-molecule compounds is a promising therapeutic avenue (Table 3).

**Table 3:** Small molecules or endogenous molecules with promising cytotoxic effect on melanoma through regulated cell death pathway other than apoptosis

RCD	Small molecules	Mechanism	Cell line/ in vivo	References
Autophagy dependent cell death	HydroxyCQ	Blocking of autophagosmoe fusion with lysosome; augmented temozolomide-induced melanoma cell death	C8161 melanoma spheroid	[55]
	7-hydroxydehydronuciferine	Cytotoxic to melanoma cell	A375.S2, A375 and A2058 melanoma cell; A375.S2 cells in mouse model	[57]
	4-PBA	Reduction of ER stress and basal autophagy; susceptibility to apoptosis induction	SK-Mel-110 *BRAF *^V600E^ cell	[51]
Pyroptosis	Doxorubicin, cisplatin, actinomycin-D, topotecan, CPT-11, etoposide and mitoxantrone	Pyroptosis induction through GSDME cleavage	GSDME ( +) SH-SY5Y neuroblastoma and MeWo skin melanoma cell	[71]
	Etoposide	Reinduction of GDEME cleavage and pyroptosis after resistance development to PLX4720 (BRAFi) and PD0325901 (MEKi)	YUMM1.7 cell from combination resistant tumor (CRT), in mouse model	[74]
	GSK2334470 and trametinib	PDPK1 and MEK inhibition to induce GSDME mediated pyroptosis, increased CD8 + T cells in tumor	WM1361A, WM1366, SK- -30 and SK -MEL-173 cell (all NRAS mutant), in mouse model	[76]
	Iron dextran	Iron-activated ROS via a Tom 20-Bax-caspase 3- GSDME pathway	A375 melanoma cell in mouse model	[77]
	Raptinal (caspase 3 activator)	Induction of pyroptosis and delay the growth of BRAFi and MEKi-resistant tumor cells in vivo	Human A375, WM35 and mouse D4M3.A and YUMM1.7 melanoma cell; YUMM1.7 and CRT47R cell in mouse model	[83]
	Temozolomide and chloroquine	Autophagy inhibition and inflammasome activation after development of BRAF inhibitor resistance	Melanoma cell from patients receiving BRAFi (vemurafenib), in mouse model	[75]
Necroptosis	Curaxin CBL0137	To trigger Z-DNA formation in melanoma-infiltrating fibroblasts to induce ZBP1-dependent necroptosis	Immune checkpoint blockade non-responsiveness mouse model (B16-F10 melanoma in *Zbp1*^*−/−*^ mouse)	[96]
	GNE684	Inhibition of cross-species RIPK1; RIPK1 inhibition by GNE684 or knock-in of *Ripk1*^*D138N/D138N*^ did not affect B16 metastasis	B16-F10 cell in mouse model	[93]
	N-TiO_2_	Impaired autophagosome -lysosome fusion, the blockade of autophagy flux via ROS production after photoactivation	A375 melanoma cell	[88]
	PK68	RIPK1 inhibition and repression of melanoma metastasis	B16-F10 cell in mouse model	[92]
Necroptosis, ferroptosis	BAY 87–2243	Mitochondrial complex I inhibition to increase ROS; reduced cell death by necrostatin/ knockdown of RIPK1 or MLKL; also ferrostatin/ knockdown of GPX4	Human G361 and SK-MEL-28 melanoma cell	[97]
Ferroptosis	Biomineralized nanovaccine with Fe^+3^ and photosensitizer IR820	Increase ROS and lipid peroxide, decreased GSH level, GPX4 inhibition, augmented anti-CTLA4 antibody efficiency	B16-OVA cell, in mouse model	[114]
	Nobiletin	GSK3β-mediated Keap1/Nrf2/HO-1 signaling pathway	SK-MEL-28 melanoma cell	[108]
	Phyto-sesquiterpene lactone, DET, and DETD-35	Lipid ROS accumulation, GPX4 inhibition	A375, A2058 and SK-MEL-2 melanoma cell, PLX4032 (vemurafenib) resistant A375 cell	[109]
Not sure, possible cuproptosis	Elesclomol	Elimination of the slow-cycling melanoma cell under the treatment of cytotoxic drugs (cisplatin)	JARID1B^high^ WM3734 melanoma cell	[134]

RCD	Endogenous molecules	Mechanism	Cell line/ in vivo	References
Autophagy dependent cell death	*CCL5*	Increased CCL5 expression in *BECN1-* melanoma, CCL5- dependent increased infiltration of NK cells to the tumor	*BECN-* B16-F10 melanoma cell in mouse model	[61, 62]
	*MAP1LC3B*	Restoration to killing by tumor-reactive T cells by this autophagy initiator	Human PTEN-silenced melanoma cell	[59]
	miR-216b	Ectopic expression increases the efficacy of vemurafenib both in vitro and in vivo	A375 and A375- vemurafenib- resistant cell, in mouse model	[56]
Pyroptosis	DNA vaccine consisting of plasmids encoding caspase-1 and an ovalbumin- derived CD8 T cell epitope	GSDMD dependent cell death, amplified antigen-specific CD8 T cell responses and longer survival	HEK-293 cell line, B16-OVA melanoma cell in C57BL/6 mouse model	[82]
Necroptosis	Tumor delivery of MLKL mRNA	Inhibition of primary and distant melanoma growth; augmented antitumor activity with immune checkpoint blockade therapy	B16-OVA melanoma cell in C57BL/6 mouse model	[95]
Ferroptosis	miR-21-3p-loaded gold nanoparticles	Enhance lipid ROS generation and ferroptosis promotion; synergistic with anti-PD-1 antibody to promote ferroptosis	A375, A2058 melanoma cell; B16-F10 cell in mouse model	[113]

The second part of this review focuses on summarizing five nonapoptotic RCDs in melanoma: autophagy-dependent cell death, pyroptosis, necroptosis, ferroptosis, and cuproptosis. Although melanoma cells show vulnerability to the induction of RCD, it is challenging to induce cells to succumb to a specific type of RCD due to the complexity and crosstalk among RCD pathways, particularly in cancer cells such as melanoma. Simultaneous regulation of multiple RCD subroutines may help overcome resistance to specific types of RCD [47]. Thus, future research should be directed at exploring the interplay between different RCD pathways, as it holds promise for melanoma treatment as a part of combination therapy with immunotherapy and in targeted therapy. In addition, prognostic models using pyroptosis, ferroptosis, and cuproptosis-related genes or lncRNAs respectively have been reported [77–79, 115, 116, 123, 125], and further collaboration may offer reliable biomarkers to treat melanoma.

Overall, a comprehensive understanding of the molecular mechanisms underlying melanoma development, combined with the exploration of novel therapeutic strategies targeting RCD pathways, shows the potential to further enhance treatment outcomes and address existing challenges in melanoma management.

## Data Availability

The datasets supporting the conclusions of this article are included within the article.

## Abbreviations

AJCC: American Joint Committee on Cancer
ATG5: Autophagy related 5
ATP7A/B: ATPase copper transporting alpha/beta
Batf3: Basic leucine zipper transcriptional factor ATF-like 3
BAX: BCL2 associated X
BNIP3: BCL2-interacting protein 3
BRAF: B-Raf proto-oncogene, serine/threonine kinase
CARD: Caspase-activation and recruitment domain
CCL5: C-C motif chemokine ligand 5
CDKN2A: Cyclin dependent kinase inhibitor 2A
c-KIT: KIT proto-oncogene, receptor tyrosine kinase
CRGs: Cuproptosis-related genes
CSD: Cumulative solar damage
CTLA4: Cytotoxic T-lymphocyte-associated protein 4
CYLD: CYLD lysine 63 deubiquitinase
DAMP: Damage-associated molecular pattern
DBT: Dihydrolipoamide branched chain transacylase E2
DLAT: Dihydrolipoamide S-acetyltransferase
DLD: Dihydrolipoamide dehydrogenase
DLST: Dihydrolipoamide S-succinyltransferase
EDNRB: Endothelin B receptor
FDX1: Ferredoxin 1
GSDMA/B/C/D/E: Gasdermin A/B/C/D/E
GCSH: Glycine cleavage system protein H
GLS: Glutaminase
GPX4: Glutathione peroxidase 4
GSH: Glutathione
GSK3β: Glycogen synthase kinase-3 beta
HMGB1: High mobility group box 1
HO-1: Heme oxygenase-1
IFN-I: Type I interferon
Keap1: Kelch-like ECH-associated protein 1
KIF14: Kinesin family member 14
LAG3: Lymphocyte-activation gene 3
LC3: Microtubule-associated protein 1A/1B-light chain 3
LIAS: Lipoic acid synthetase
LIPT1: Lipoyltransferase 1
L-O: Lipid alkoxy radicals
L-OH: Lipid alcohols
L-OOH: Lipid hydroperoxides
MAP1LC3B: Microtubule associated protein 1 light chain 3 beta
MAPK: Mitogen-activated protein kinase
MDSC: Myeloid-derived suppressor cells
MEK: Mitogen-activated protein kinase kinase
MLKL: Mixed lineage kinase domain-like
MTF1: Metal response element-binding transcription factor 1
N-TiO_2_: Nitrogen-doped titanium dioxide
NF1: Neurofibromin 1
NRAS: NRAS proto-oncogene, GTPase
NRF2: Nuclear factor erythroid 2-related factor 2
OXPHOS: Oxidative phosphorylation
PD-1: Programmed cell death protein 1
PDHA1: Pyruvate dehydrogenase E1 component subunit alpha 1
PDHB: Pyruvate dehydrogenase E1 component subunit beta
PI3K: Phosphoinositide-3-kinase
PRRs: Pattern recognition receptors
PTEN: Phosphatase and tensin homolog
PTPRC: Protein tyrosine phosphatase receptor-type C
PARP1: Poly (ADP-ribose) polymerase 1
RIPK1/3: Receptor-interacting protein kinase 1/3
SEER: Surveillance, Epidemiology, and End Results
SHARPIN: Shank associated RH domain interactor
SLC31A1: Solute carrier family 31 member 1
SLC39A6: Solute carrier family 39 member 6
SLC3A2: Solute carrier family 3 member 2
SLC7A11: Solute carrier family 7 member 11
SQSTM1: Sequestosome 1
TERT: Telomerase reverse transcriptase
TMB: Tumor mutation burden
TMEM117: Transmembrane protein 117
ULK1: Unc-51-like kinase 1
UVRAG: UV radiation resistance-associated gene
ZBP-1: Z-form nucleic acid binding protein 1
